# Astrocytic Coverage of Dendritic Spines, Dendritic Shafts, and Axonal Boutons in Hippocampal Neuropil

**DOI:** 10.3389/fncel.2018.00248

**Published:** 2018-08-17

**Authors:** Nikolay Gavrilov, Inna Golyagina, Alexey Brazhe, Annalisa Scimemi, Vadim Turlapov, Alexey Semyanov

**Affiliations:** ^1^UNN Institute of Neuroscience, N. I. Lobachevsky State University of Nizhny Novgorod, Nizhny Novgorod, Russia; ^2^Department of Biophysics, Faculty of Biology, M. V. Lomonosov Moscow State University, Moscow, Russia; ^3^Department of Biology, University at Albany, The State University of New York (SUNY), Albany, NY, United States; ^4^Institute of Information Technologies, Mathematics and Mechanics, N. I. Lobachevsky State University of Nizhny Novgorod, Nizhny Novgorod, Russia; ^5^Shemyakin-Ovchinnikov Institute of Bioorganic Chemistry, Russian Academy of Sciences, Moscow, Russia; ^6^All-Russian Research Institute of Medicinal and Aromatic Plants, Moscow, Russia

**Keywords:** astrocyte, branches, leaflets, dendritic shaft, axonal bouton, spatial entropy, spatial complexity

## Abstract

Distal astrocytic processes have a complex morphology, reminiscent of branchlets and leaflets. Astrocytic branchlets are rod-like processes containing mitochondria and endoplasmic reticulum, capable of generating inositol-3-phosphate (IP_3_)-dependent Ca^2+^ signals. Leaflets are small and flat processes that protrude from branchlets and fill the space between synapses. Here we use three-dimensional (3D) reconstructions from serial section electron microscopy (EM) of rat CA1 hippocampal neuropil to determine the astrocytic coverage of dendritic spines, shafts and axonal boutons. The distance to the maximum of the astrocyte volume fraction (VF) correlated with the size of the spine when calculated from the center of mass of the postsynaptic density (PSD) or from the edge of the PSD, but not from the spine surface. This suggests that the astrocytic coverage of small and larger spines is similar in hippocampal neuropil. Diffusion simulations showed that such synaptic microenvironment favors glutamate spillover and extrasynaptic receptor activation at smaller spines. We used complexity and entropy measures to characterize astrocytic branchlets and leaflets. The 2D projections of astrocytic branchlets had smaller spatial complexity and entropy than leaflets, consistent with the higher structural complexity and less organized distribution of leaflets. The VF of astrocytic leaflets was highest around dendritic spines, lower around axonal boutons and lowest around dendritic shafts. In contrast, the VF of astrocytic branchlets was similarly low around these three neuronal compartments. Taken together, these results suggest that astrocytic leaflets preferentially contact synapses as opposed to the dendritic shaft, an arrangement that might favor neurotransmitter spillover and extrasynaptic receptor activation along dendritic shafts.

## Introduction

Synaptic contacts are highly specialized neuronal compartments. The presynaptic terminal is an axonal varicosity that is closely apposed to the postsynaptic terminal. The dendritic spine is the postsynaptic terminal of a glutamatergic synapse. Dendritic spines are highly dynamic structures that can be classified into several groups based on their morphology (e.g., mushroom spines, thin spines etc.) (Hering and Sheng, 2001). Spines have a neck and a head with an electron dense PSD. The space between synapses is occupied by astrocytic processes (Kettenmann et al., 1996; Chao et al., 2002; Witcher et al., 2007; Verkhratsky and Nedergaard, 2018).

The intimate anatomical and physiological interaction between spines, boutons and perisynapstic astrocytic process (PAP) has been referred to as the “synaptic triate” (Kettenmann et al., 1996), “tri-partite synapse” (Araque et al., 1999), or “astrocytic cradle” (Nedergaard and Verkhratsky, 2012; Verkhratsky and Nedergaard, 2014). PAPs have a high density of glutamate transporters and of inward rectifying K^+^ channels. They are thought to shape both synaptic transmission and extrasynaptic signaling via glutamate buffering/uptake and K^+^ clearance, respectively (Walz, 2000; Danbolt, 2001; Chever et al., 2010; Sibille et al., 2014). Astrocytic uptake limits both glutamate escape out of the synaptic cleft (Rusakov and Kullmann, 1998; Diamond, 2001; Huang and Bordey, 2004; Rose et al., 2018) and its diffusion into the synaptic cleft (Lozovaya et al., 2004; Wu et al., 2012). K^+^ clearance reduces local, activity-dependent extracellular K^+^ accumulation, which can depolarize the presynaptic terminal and facilitate the neurotransmitter release (Poolos et al., 1987; Shih et al., 2013; Sibille et al., 2014; Contini et al., 2017; Lebedeva et al., 2018). PAPs also contain glycogen granules and can metabolically interact with neighboring synapses (Phelps, 1972; Koizumi, 1974; Calì et al., 2016). Astrocytic glycogen is converted into lactate and delivered to neurons through monocarboxylate transporters (MCTs) to be used as an energy substrate (Quistorff et al., 2008; Matsui et al., 2017). This exchange of metabolites, commonly referred to as the “astrocyte-neuron lactate shuttle” (ANLS), is upregulated during sleep deprivation and exhaustive exercise (Magistretti, 2011; Matsui et al., 2017). In addition to the energy substrate function, lactate released by astrocytes can serve as a signaling molecule/gliotransmitter to excite neurons and to modulate astrocytic Ca^2+^ activity (Tang et al., 2014; Lebedeva et al., 2015; Magistretti and Allaman, 2018). Astrocytes release several other gliotransmitters such as D-serine, glycine, glutamate, GABA, ATP, adenosine etc (Zorec et al., 2012; Araque et al., 2014). Astrocytic D-serine facilitates synaptic plasticity (Panatier et al., 2006; Henneberger et al., 2010; Papouin et al., 2017). Glutamate activates presynaptic metabotropic (Semyanov and Kullmann, 2000; Liu et al., 2004b) and axonal kainate receptors (Semyanov and Kullmann, 2001; Liu et al., 2004a), which in turn shape the profile of neurotransmitter release (Kullmann and Semyanov, 2002; Gordleeva et al., 2012). GABA release from astrocytes might contribute to increase of tonic GABA_A_ receptor activation in neurons (Semyanov et al., 2004; Angulo et al., 2008; Héja et al., 2012). Despite the wealth of physiological data suggesting multiple types of interaction between the synapse and PAPs, there is no firm anatomical evidence for the existence of particular types of PAPs belonging to a particular type of “tri-partite” synapse. Our general understanding, based on 3D EM reconstructions in the hippocampal neuropil, is that astrocytes are sponge-like structures just filling all the space between synapses, dendrites, axons, etc. (Witcher et al., 2007; Patrushev et al., 2013; Medvedev et al., 2014). Nevertheless, in the rodent hippocampus the astrocyte VF is larger around dendritic spines than axonal boutons (Lehre and Rusakov, 2002). There is, however, an ongoing contentious. Although some ultrastructural studies indicate that there is a higher density of astrocytic processes around large mushroom spines (Jones and Greenough, 1996; Genoud et al., 2006; Lushnikova et al., 2009; Bernardinelli et al., 2014a,b), other works do not confirm these results, possibly due to the use of different analytical approaches (Medvedev et al., 2014; Heller and Rusakov, 2015). For this reason, a more detailed structural analysis of astrocytic coverage at hippocampal synapses is needed. Recent reports have indicated that astrocytic processes are heterogeneous and can be distinguished into thin organelle-free structures (leaflets) and thick processes that host endoplasmic reticulum (ER) and mitochondria (larger branches, smaller branchlets, and perivascular endfeet) (Patrushev et al., 2013; Khakh and Sofroniew, 2015).

Here we study how astrocytic leaflets and branchlets distributed around different types of dendritic spines, dendritic shafts and axonal boutons.

## Materials and methods

### Sample preparation and electron microscopy

Wistar rats (male, 250 ± 25 g) were transcardially perfused with a mixture of 3% paraformaldehyde and 0.5% glutaraldehyde in 0.1 M Na-cacodylate buffer (pH 7.2–7.4). The perfused brains were cut into 150 μm thick slices, which were fixed with 2.5% glutaraldehyde for 24 h and impregnated with 1% osmium tetroxide and 0.01% potassium dichromate for 1–2 h. The slices were then dehydrated in graded aqueous solutions of ethanol from 40 to 96% (10 min each) and finally in 100% acetone (three washes, 10 min each). Dehydrated slices were then incubated in a mixture of 50% epoxy resin and 50% pure acetone for 30 min. They were then embedded in a capsule of pure epoxy resin (Epon 812/AralditeM) at 60°C for 1 h and polymerized overnight at 80 °C.

Serial sections (60–70 nm) of CA1 *str.radiatum* were prepared using a Diatome diamond knife and were collected using Pioloform-coated slot copper grids. Sections were counterstained with saturated ethanolic uranyl acetate, followed by lead citrate, and then placed in a grid holder. Images were obtained with a JEOL 1010 EM at 6000x magnification.

EM negatives were scanned at 1200 dpi resolution. The scanned images were aligned using custom-made software. Complete reconstruction of astrocytic processes, dendrites, dendritic spines, PSDs and axons was performed using the software Reconstruct (by Dr. John Fiala, provided free of charge at SynapseWeb, Kristen M. Harris, PI) after manually tracing the contours of these structures (Supplementary Figure 1). The meshes generated by the Reconstruct software served as input data for our morphological analysis. The total volume of the reconstructed block was 393 μm^3^.

### Equidistant surface analysis

PSDs, dendritic spines, axonal boutons and dendritic shaft chunks, were enclosed by a triangulated sphere for the equidistant surface analysis. The sphere was then transformed into the equidistant surface at a given distance (*d*) (Supplementary Figure 2): We calculated the shortest distance between the object and each vertex of the triangulated sphere. If this distance was smaller than *d*, then the vertex was moved away from the object to the distance equal to *d*. If this distance was larger than *d*, then the vertex was moved closer to the object and to the distance equal to *d*. Rearranging all the vertexes of the triangulated sphere gave a satisfactory approximation of the equidistant surface.

For astrocytic coverage analysis, equidistant surfaces were plotted (1) around the center of mass of the PSD, (2) around the edges of the PSD or (3) around the spine surface. In the first case, the equidistant surfaces were concentric spheres with *d* = 0 at the PSD center of mass. In the second case, *d* = 0 was at the edge of the PSD. In the third case, *d* = 0 was at the side spine surface: First, the equidistant surfaces were plotted around the entire reconstructed spine. Then parts of these surfaces which were inside presynaptic half-space and inside dendritic half-space were excluded (Supplementary Figure 2).

Dendritic shafts could be were recognized as long objects striding the whole reconstructed block. Equidistant surfaces were not generated around whole dendritic shafts because this would produce spatial averaging of their uneven microenvironment. Therefore, dendritic shafts were split into chunks of 1 μm length, which were treated as separate objects, and equidistant surfaces were plotted around their membrane-covered parts.

Axonal boutons radii were calculated as described in Supplementary Figure 2C.

### Diffusion simulations

Glutamate diffusion from synapses of different radii was modeled using Matlab 2016b (Mathworks; Natick, MA). We positioned one synapse at the center of a 10 μm^3^ world (Diamond, 2005). The pre- and post-synaptic terminals were represented as two hemispheres separated by a 20-nm thick synaptic cleft and surrounded by a 50 nm-wide extrasynaptic region. The radius of the PSD was calculated as 1/3 of the sum of the synapse radius and the extrasynaptic region. A perisynaptic annulus separated the PSD from the extrasynaptic region. The width of the perisynaptic region was calculated by subtracting the PSD area from the area covered by the synaptic and extrasynaptic regions. At the beginning of each simulation, we released 2,000 glutamate molecules from a point source located at the center of the synaptic cleft. Each glutamate molecule diffused with an apparent diffusion coefficient D^*^ = 0.33 μm^2^/ms, according to the experimental measures and theoretical predictions at 36–37°C (Nielsen et al., 2004). Outside the cleft, D^*^ was reduced to account for the tortuosity of the hippocampal neuropil, measured experimentally in P14-21 mice (λ = 1.45) (Scimemi et al., 2009). The space surrounding the extrasynaptic volume contained glutamate transporters at a concentration of 20 μM (Lehre and Danbolt, 1998), each of which was represented using a simplified Markov model of the glial glutamate transporter GLT1 (Bergles et al., 2002; Diamond, 2005; Scimemi et al., 2009). All reaction rates were corrected for Q_10_ = 3 to account for the temperature dependence of the glutamate transport process. The extracellular space was subdivided into 10-nm thick concentric shells and the simulations were run with a time step of 1 μs for a total of 10,000 steps (10 ms). At each time step, we calculated the number of glutamate molecules in the volume of the synaptic cleft above the PSD area and in the 50 nm-wide extrasynaptic volume. The obtained glutamate concentration waveforms were used to drive GluN (Lester and Jahr, 1992) and GluA Markov models (Jonas, 1993) in ChanneLab2 (Synaptosoft; Decatur, GA). The number of glutamate molecules bound by glutamate transporters were used to calculate the time course of the STCs.

The source code for these simulations is available at https://sites.google.com/site/scimemilab2013/software.

### Complexity-entropy spectrum analysis

The goal of the complexity-entropy analysis was to quantify the spatial properties of representative 2D images with respect to their balance between randomness and structural order, triviality and complexity. In mathematical terms, this is described as location in the complexity-entropy plane or their distribution along a complexity-entropy spectrum. Highly ordered structures (e.g., a regular grating) have near-zero entropy and near-zero complexity. In contrast, completely disordered structures (e.g., independent and identically distributed Gaussian samples) have maximal entropy and small statistical complexity. Intermediate values of entropy are associated with higher values of complexity if the underlying pattern contains features with preferred orientation. For example, signals generated by systems with deterministic chaos result in a complexity-entropy spectrum with near-0.5 peak complexity and near-0.7 entropy (Lamberti et al., 2004; Rosso et al., 2007).

Below we provide a concise description of the complexity-entropy analysis theory and implementation. More in-depth theoretical overview on the entropic complexity measures can be found in (Lamberti et al., 2004; Martin et al., 2006; Rosso et al., 2007) and the implementation is described in a method paper (Brazhe, 2018). The implementation code is available at DOI:10.5281/zenodo.1217636.

Complexity and entropy measures can be based on a feature distribution of the analyzed patterns, which can be compared to equiprobable or singular feature cases. Accordingly, the relative entropy (*H*) was defined as the Shannon entropy:

(1) $$\begin{array}{r} H\left[P\right]:=S\left[P\right]/S_{\text{max}}=\left(-\overset{N}{\underset{i=1}{\sum }}P_{i}log_{2}P_{i}\right)/log_{2}N, \end{array}$$

normalized by the entropy of an equally probable distribution (*S*_max_), thus giving values in the range [0 … 1]. Here *N* is the number of features analyzed. The complexity measures were based on the notion of disequilibrium (Lamberti et al., 2004) and defined in terms of normalized Jensen-Shannon divergence from an equally probable distribution (Rosso et al., 2007):

(2) $$\begin{array}{r} C\left[P\right]:=H\left[P\right]J\left[P,P_{e}\right]/J_{\text{max}}, \end{array}$$

In this expression, *J*[*P,P*_*e*_] is Jensen-Shannon divergence of some distribution P from an equally probable distribution *P*_*e*_:

(3) $$\begin{array}{r} J\left[P,P_{e}\right]=S\left[\frac{P+P_{e}}{2}\right]-\frac{1}{2}\left(S\left[P\right]+S\left[P_{e}\right]\right), \end{array}$$

and *J*_max_ is obtained when the probability of just one feature is equal to 1, while the probability of all other features is zero.

Armed with the probability-based definitions of entropy and complexity, one needs a way to build descriptive feature probabilities from spatial patterns, preferably allowing for multiscale resolution and local analysis. A spatial pattern can be described in terms of local edge orientations and scales. Thus, statistics of the shearlet transform coefficients appears as a promising probability-inducing image decomposition (Kutyniok and Labate, 2012). Shearlets are a novel image analysis tool, an orientation-aware analog of wavelets for multivariate data. In short, the shearlet transform separates an input image into a spectrum of local spatial scales and orientations. Thereafter, we treated normalized power of shearlet coefficients as probability densities of given spatial feature orientation and scale at a given location.

The complexity-entropy analysis is illustrated on a toy example of a gradually corrupted stripe pattern in Supplementary Figure 3. Before introducing noise, the spatial pattern is ordered and corresponds to low entropy–low complexity region of the spectrum. Disrupting the image by swapping intensity values with between neighboring pixels increases entropy and is accompanied by a transient rise in complexity due to changes in the distribution of the power of shearlet coefficients.

We performed the complexity-entropy spectrum analysis of the spatial organization of leaflets and branchlets separately. Segmentation images (i.e., images with pixel values set to one within identified leaflets or branchlets or to zero otherwise) were sum-projected along the Z-axis and down-sampled 2 times in the X and Y directions to be treatable by the shearlet transform library (Gregory R. Lee, https://github.com/grlee77/PyShearlets). The resulting images represented the abundance of the given astrocytic structure in each point. Entropy and complexity calculations were only performed in regions containing non-zero pixels, while other areas (black in Figure 5A) were masked out.

The resulting pairs of H and complexity (C) values at each pixel were then binned and mapped onto the complexity-entropy plane (Figure 5B). To condense the apparent differences between the spectra of branchlets and leaflets spatial patterns, we plotted a “median curve,” which represents median complexity for all (branchlets + leaflets) points at each entropy value. This was achieved by binning all points into 51 intervals of entropy values, calculating the median complexity values for each interval, and then fitting the resulting set of points with a smooth cubic spline. This allowed us to use an “excess complexity” parameter to describe each (H,C) point by a single value of signed deviation from the “median curve.” The resulting distributions of excess complexity (kernel density estimates) for branchlets and leaflets spatial patterns are shown in Figure 5C.

The results of the presented complexity/entropy analysis can potentially depend on the orientation of the astrocyte relative to the imaged block. Astrocytes are 3D structures, and using a 3D measure of complexity and entropy might provide more information than analysis of projections along one axis. However, increasing dimensionality could deteriorate the shearlet-based definition of spatial feature probabilities because higher degrees of freedom would demand larger samples. Ideally, if resolution is equal along all three axes (X-Y-Z), one could draw a plane corresponding to the two first principal axes of the cloud of astrocytic voxels, and project all structures to this principal plane or to a set of planes, parallel to the principal one. However, in the case of serial section EM, Z-axis resolution is typically lower that X-Y resolution. Therefore, only the projection along Z-axis is possible.

### Statistical analysis

The data are presented as mean ± standard error of mean (SEM). The statistical difference was tested with Mann–Whitney test using Origin 8 (OriginLab Corp.). The level of significance was set at *P* < 0.05.

## Results

### Astrocytic coverage of thin and mushroom spines

**Figure 1:**
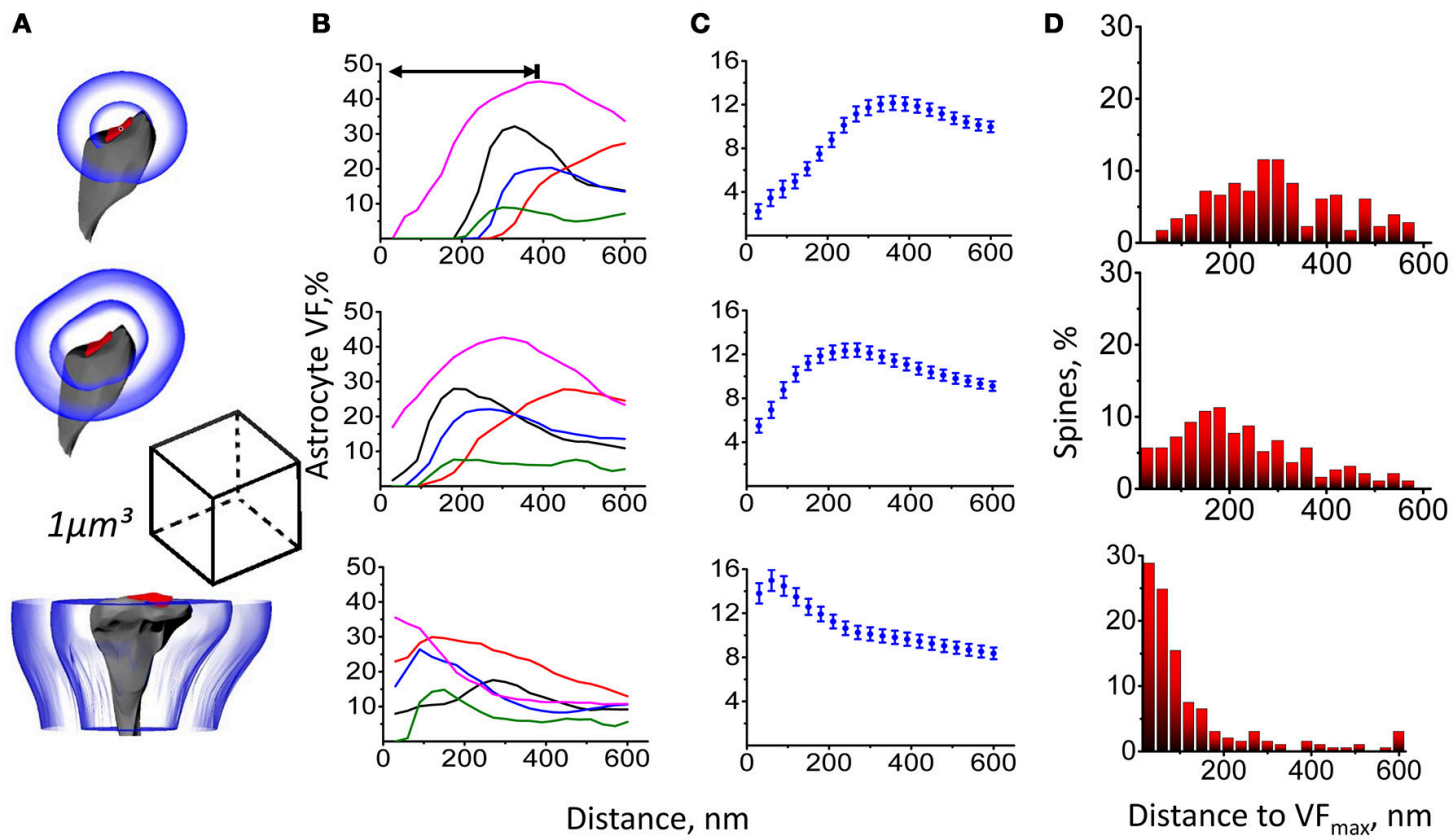
Astrocyte VF distribution around dendritic spines. **(A)** Astrocyte VF was measured between equidistant surfaces constructed around PSD center (top), around PSD surface (middle) and around side surfaces of the dendritic spine (bottom). **(B)** Changes of astrocyte VF with the distance from PSD center (top), from PSD surface (middle) and from spine surface (bottom) for 5 individual spines. Double-sided arrow indicates the distance to the VF_max_. **(C)** Summary data as in **(B)** are presented as mean ± SEM for 207 spines. **(D)** Percentage of dendritic spines with VF_max_ at a different distance from PSD center (top), PSD surface (middle), and spine surface (bottom). The bin size of 30 nm was equal to the distance step in **(A–C)**.

Serial EM sections were used to generate a full 3D reconstruction of a 393 μm^3^ block from hippocampal *str.radiatum*. Astrocytic processes, dendritic shafts, dendritic spines, axons and axonal varicosities were visually identified, manually traced and 3D rendered. We analyzed the spatial distribution of astrocyte VF around dendritic spines using three different approaches (Figure 1A). (1) We calculated the astrocyte VF at increasing distances from the center of mass of the PSD (similar to the approach used by Medvedev et al., 2014). (2) We calculated the astrocyte VF at increasing distances from the edge of the PSD (similar to the approach used by Patrushev et al., 2013). (3) We calculated astrocyte VF at increasing distances from the surface of the dendritic spine (without including the volume occupied by the presynaptic half-space and dendritic shaft half-space). The first approach estimates the distribution of astrocyte around the presynaptic glutamate release site, which is closely apposed to the center of mass of the PSD. This method does not exclude the portion of the cleft around the PSD, where no astrocyte is typically present. Because synapses vary in size, the astrocyte-free area also varies with the size of the synapse. Therefore, astrocytic processes are located further away from the centers of larger synapses, suggesting these might have fewer astrocytic processes around them (Figures 1B,C). The second approach works around the fact that larger spines have larger PSDs, but the method is still prone to errors because the PSD does not cover the entire apposition zone, which is also bigger at bigger spines (Figures 1B,C). Thus, both approaches provide a measure of astrocyte VF without completely excluding the volume occupied by the synapse. In contrast, the third approach provides a genuine measure of astrocyte VF around dendritic spines. As expected, our estimates for the distance from the spine to the maximum astrocyte VF (VF_max_) varied depending on the method of analysis. When using the first method, the distance to VF_max_ depended on the spine head radius (Figure 1D and Supplementary Figure 4). For the second method, this trend was less pronounced. For the third method, there was no correlation between the distance to VF_max_ and the spine radius. Specifically, 28.9% of spines had VF_max_ in immediate apposition to their membrane as if they formed as “astrocytic cradle” rather than a non-specific filling of the extracellular space (Verkhratsky and Nedergaard, 2014).

**Figure 2:**
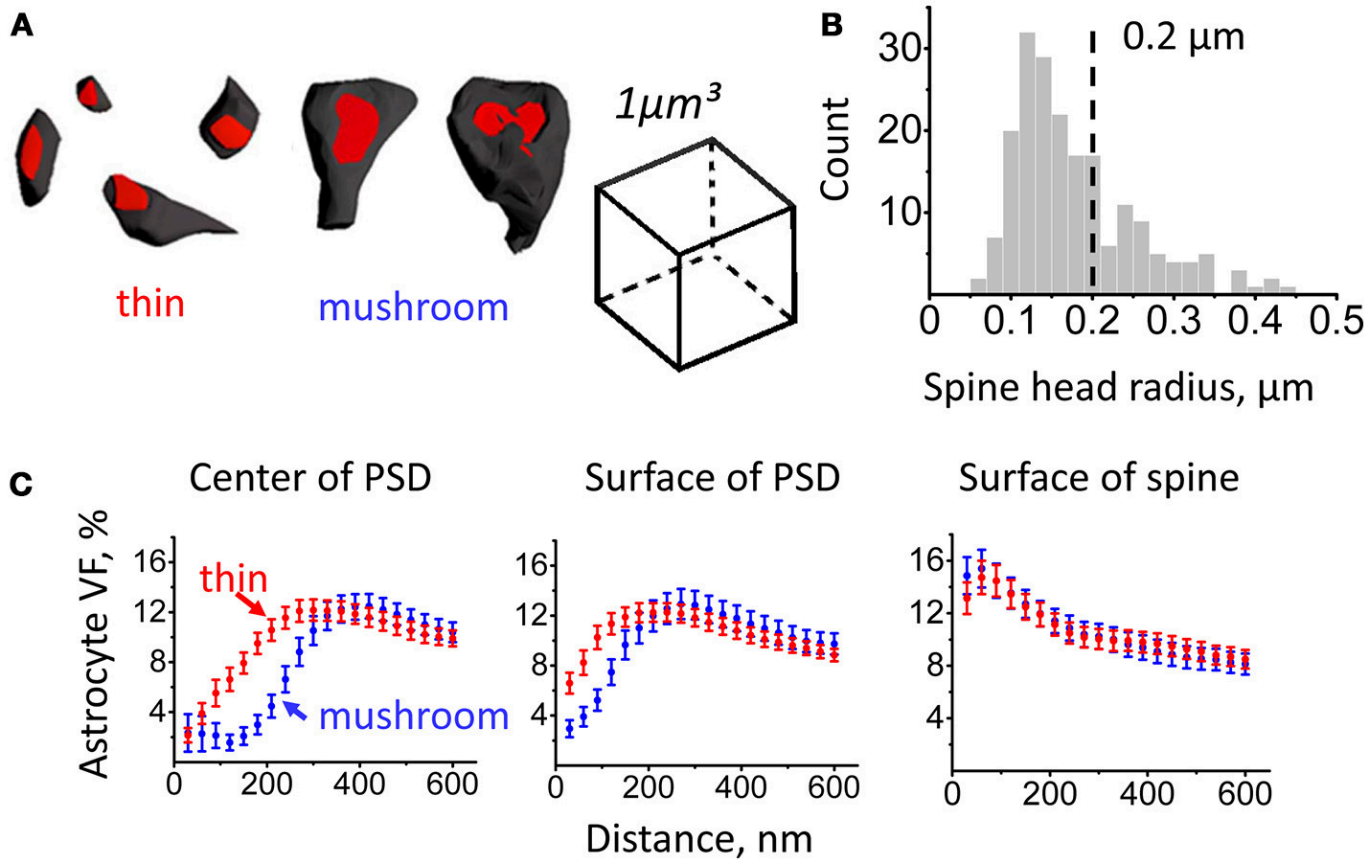
Astrocytic coverage of mushroom and thin spines. Segmentation of mushroom and thin spines was based on spine head radius. **(A)** Examples of thin and mushroom spines. **(B)** Distribution of spines according to their head radii. Spine types were segmented by the radius of 0.2 μm: mushroom spines were defined as spines with radius ≥ 0.2 μm, thin spines were defined as spines with radius < 0.2 μm. **(C)** Changes of astrocyte VF with the distance from PSD center (left), from PSD surface (middle) and from spine surface (right) for thin (red circles) and mushroom (blue circles) spines. The data are presented as mean ± SEM.

Based on morphological and physiological properties, dendritic spines can be subdivided into several classes (e.g., mushroom spines, thin spines) (Hering and Sheng, 2001). In our analysis, we considered spines with head radius < 0.2 μm as thin spines and spines with head radius > 0.2 μm as mushroom spines (Figures 2A,B). We then plotted astrocyte VF at increasing distances for spines of different sizes (Figure 2C).

Consistent with the above result, VF at a given distance (100 nm) from the center of mass of the PSD or from the edge of the PSD was larger at thin spines than at mushroom spines (from the center of mass: 5.5 ± 1.1% at thin spines, *n* = 128, and 2.2 ± 0.9% at mushroom spines, *n* = 79; *p* < 0.001 Mann–Whitney test; from the edge: 10.3 ± 0.9% at thin spines, *n* = 128, and 5.2 ± 0.8% at mushroom spines, *n* = 79; *p* < 0.001 Mann–Whitney test). In contrast, there was no significant difference in astrocyte VF at 100 nm from the spine surface between thin and mushroom spines (14.2 ± 1.2% at thin spines, *n* = 128, and 14.6 ± 1.4% at mushroom spines, *n* = 79; *p* = 0.44 Mann–Whitney test). This result suggests that the astrocytic coverage does not depend on the spine size. This finding, however, does not rule out that activity-dependent differences in the astrocytic coverage of spines might occur within each morphological group.

### Functional implications for extrasynaptic receptor activation

**Figure 3:**
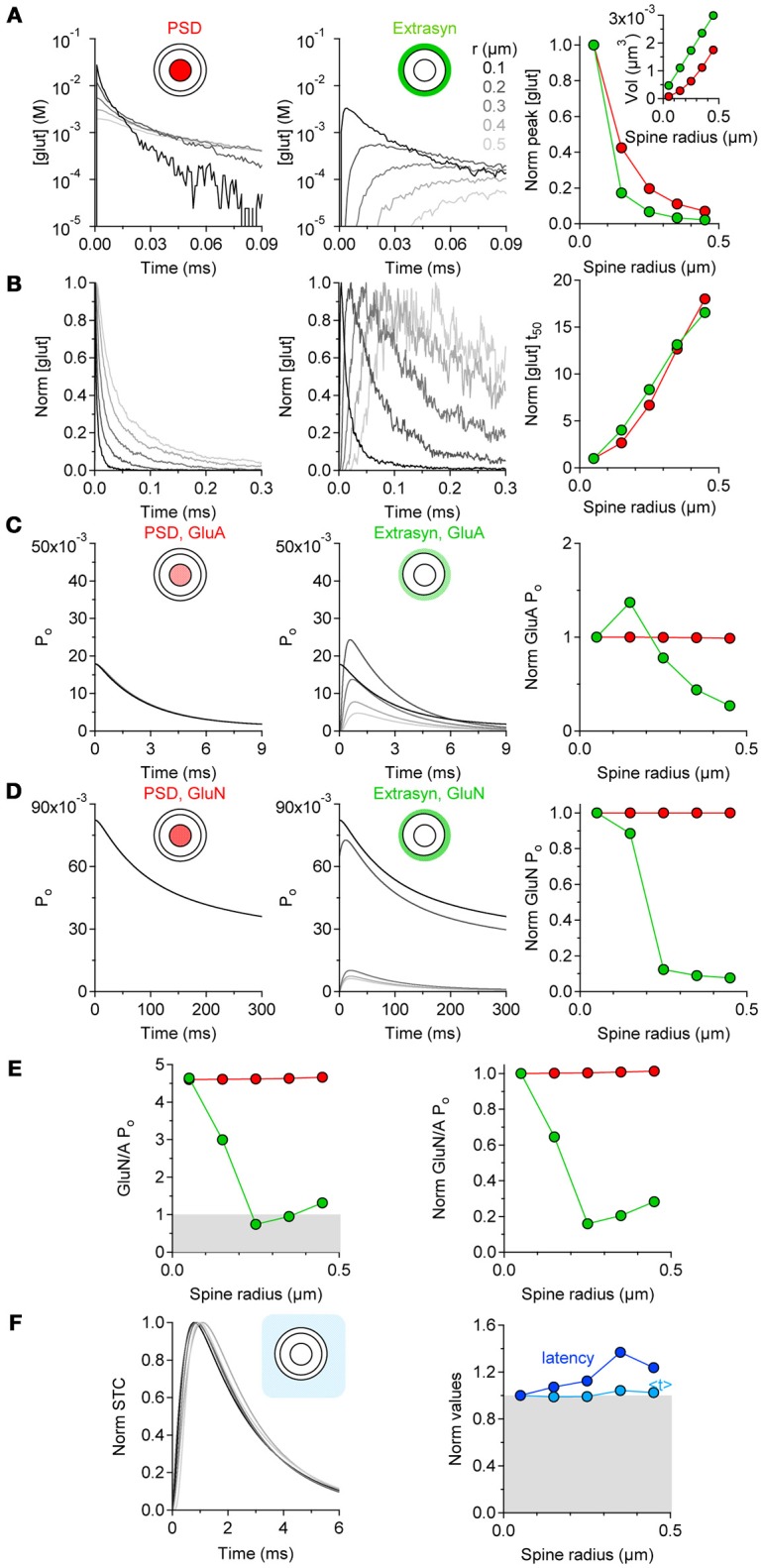
Extrasynaptic GluN activation is favored at smaller spines. **(A)** Time courses of the glutamate concentration in the volume of the synaptic cleft above the PSD (red, left panel) and in the extrasynaptic region (green, middle panel), at synapses of increasing radius (*r* = 0.05–0.45 μm). Right panel: summary graph, showing the progressively smaller peak glutamate concentration in the cleft and in the extrasynaptic region of synapses of increasing r (inset). **(B)** Peak-normalized time courses of the glutamate concentration shown in **(A)**. Right panel: summary graph showing the increased decay time of the glutamate concentration profile at larger synapses. **(C)** Open probability (P_o_) of GluA receptors in thePSD and extrasynaptic region. Right panel: summary graph of the GluA P_o_ normalized by its value at *r* = 0.05 μm. The GluA P_o_ in the PSD does not change at small and large synapses (red). In contrast, the P_o_ of extrasynaptic GluA receptors (green) is larger at small than large synapses. **(D)** GluN P_o_ in the PSD and extrasynaptic region. Right panel: summary graph of the GluN P_o_, normalized by its value at *r* = 0.05 μm. The GluN P_o_ in the PSD region does not change at small and large synapses (red). In contrast, the P_o_ of extrasynaptic GluN receptors (green) decreases abruptly as the synaptic radius increases. **(E)**. Ratio of the GluN and GluA P_o_ at synapses with different size. The GluN P_o_ is larger than the GluA P_o_ in and out of the PSD at small synapses. A reduction in the Po of GluN vs. GluA receptors occurs as the synaptic radius increases. Right panel: summary graph of the GluN/A P_o_ normalized by its value at *r* = 0.05 μm. There is a progressive reduction in the relative P_o_ of GluN and GluA receptors at larger synapses. **(F)** Peak normalized time course of synaptically-activated transporter currents (STCs) at astrocytes surrounding an active synapse with increasing radius. Right panel: summary graph of the STC latency and centroid (< t>) normalized by their values at synapses with *r* = 0.05 μm. A delay in the onset of the STC occurs at large synapses. This effect is not associated with changes in the time course of the STC.

If the level of astrocytic coverage does not scale with the spine size, what effect does this have on the activation of synaptic and extrasynaptic GluA and GluN receptors? To address this question, we performed computer simulations of glutamate diffusion. The pre- and postsynaptic terminals were represented as two hemispheres separated by a 20-nm thick synaptic cleft surrounded by a 50-nm wide extrasynaptic region. The synaptic radius (r) was varied from 50 nm to 450 nm and the radius of the PSD from 17 to 150 nm, to account for the presence of larger PSD areas at bigger spines (Ventura and Harris, 1999). We measured the time course of the glutamate concentration in the volume of the cleft above the PSD area and in the extrasynaptic volume, and used these measures to determine the open probability of GluA/N receptors and the time course of glutamate transporter current. The peak glutamate concentration became progressively smaller and prolonged in its rise and decay time at increasing values of r (Figures 3A,B), as the volume above the PSD and extrasynaptic regions increased (Figure 3A right panel, inset). The open probability of GluA and GluN receptors in the PSD did not vary appreciably among spines of different dimensions (Figures 3C,D, left and right panels). In contrast, the open probability of these receptors in the extrasynaptic region varied profoundly between small and larger spines (Figures 3C,D, middle and right panels). The open probability of extrasynaptic GluA receptors peaked at *r* = 0.15 μm and then progressively decreased (Figure 3C, right panel). GluN receptors have a higher steady-state affinity for glutamate with respect to GluA receptors (Tang et al., 1989; Trussell and Fischbach, 1989; Patneau and Mayer, 1990) (Lester et al., 1990). Their peak open probability was generally higher than that of GluA receptors (Figure 3D, middle panel), but decayed more abruptly in the extrasynaptic region of larger spines. Consequently, there was a progressive reduction in the relative activation of GluN versus GluA receptors in extrasynaptic space of bigger spines (Figure 3E). Glutamate clearance by glial transporters was delayed at larger spines, but its time course (measured as the centroid of synaptically-activated transporter currents, < t>) was similar to the one measured at smaller spines (Figure 3F). In the hippocampus, the activation of GluN receptors is crucial for the induction of long-term potentiation and depression (LTP and LTD, respectively). LTP/LTD expression is associated with spine enlargement/shrinkage, respectively (Lang et al., 2004; Nägerl et al., 2004; Zhou et al., 2004). Therefore, our findings suggest that the increased extrasynaptic GluN activation at the smaller spines might render these synapses more prone to express synaptic plasticity compared to larger spines.

### Analysis of astrocytic branchlets and leaflets

**Figure 4:**
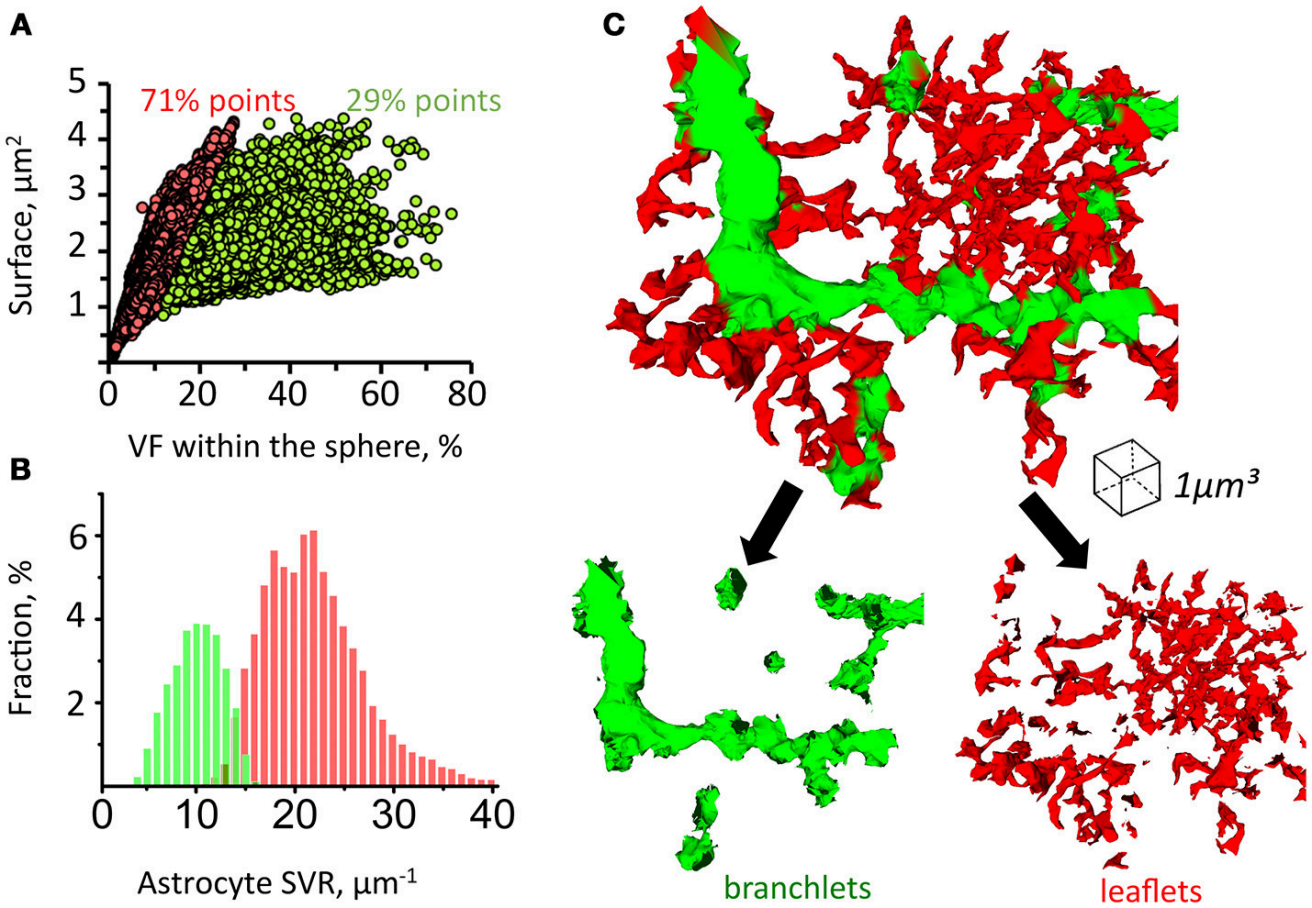
Method for sorting of astrocytic branchlets and leaflets. **(A)** Secant sphere with a 600-nm radius centered at each point on the astrocyte surface was used. Surface and VF of the astrocyte process within this sphere was calculated. The relationship between surface and VF was plotted as circles for individual points on astrocyte surface. The circles were divided into two clusters with *K*-means algorithm. Circles to the left (71% of all points) were marked in red, circles to the right (29% of all points) were marked in green. **(B)** Distribution of “green” and “red” marked astrocyte processes according to their SVR. **(C)** 3D reconstruction of astrocyte processes marked in green and red according to the classification presented in **(A,B)**. The green marked processes predominantly represent astrocytic branchlets, while the red marked processes represent leaflets.

Since astrocytic leaflets are thin and flat, while branchlets are rod-like structures, they can be distinguished according to their surface-to-volume ratio (SVR) (Patrushev et al., 2013). To establish local SVRs we employed previously suggested secant spheres method (Patrushev et al., 2013). Following this method, a 300 nm-radius sphere is was centered along each point of the astrocyte surface. The we analyzed the volume and the surface area of astrocytic chunks confined inside of the sphere. We plotted the relation between the surface area and the volume of each chunk and used a K-means algorithm to identify two clusters of points (Figure 4A). This method allowed us to satisfactorily separate leaflets and branchlets according to their SVR distributions (Figures 4B,C and Supplementary Video).

**Figure 5:**
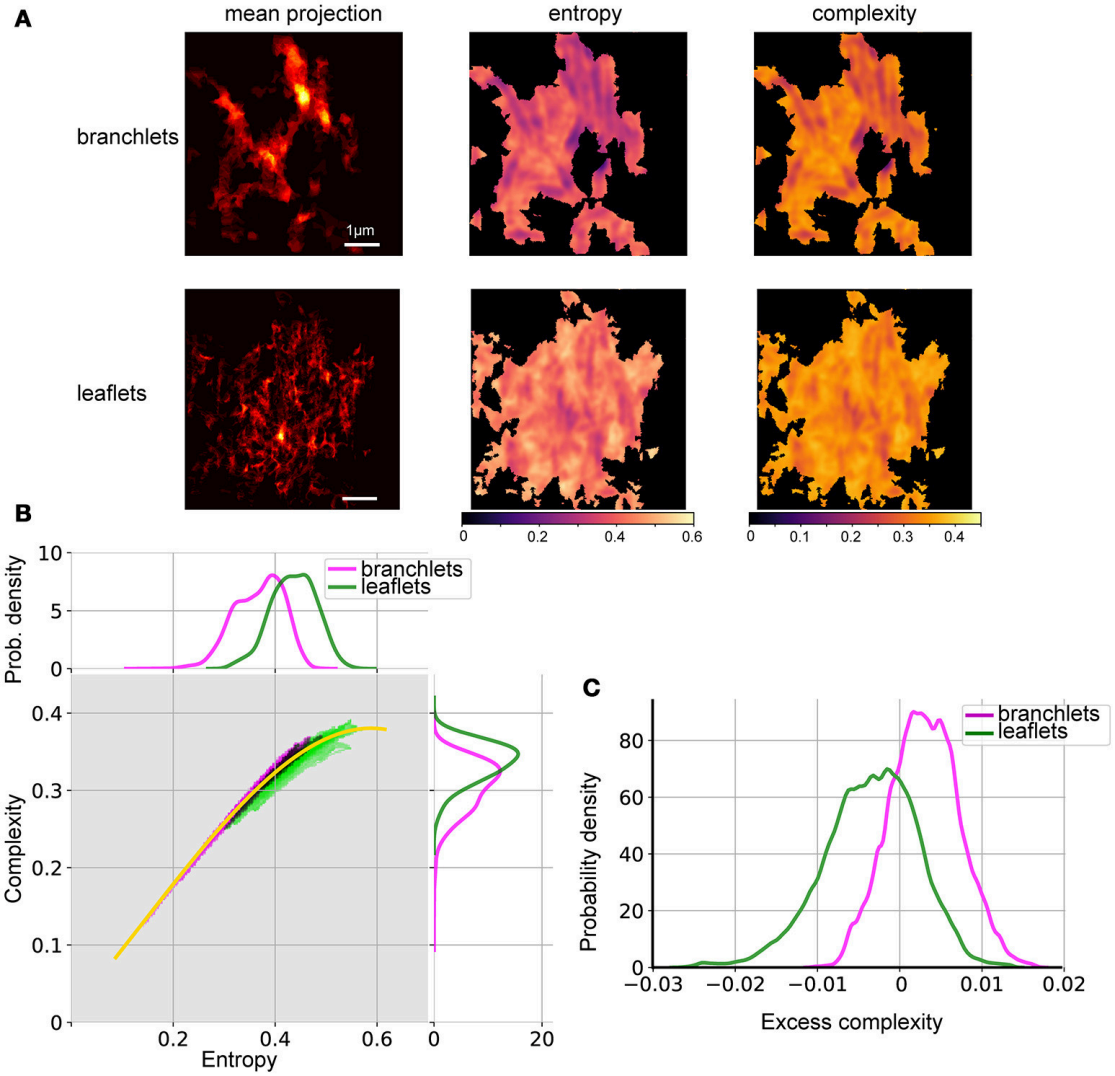
Spatial entropy and complexity of astrocytic processes. **(A)** Mean projection of segmented astrocytic processes (left), spatial entropy (middle), and spatial complexity (right) for astrocytic branchlets (top) and leaflets (bottom). Areas not containing any structures were assigned 0 entropy and complexity. **(B)** Middle, distributions of complexity and entropy for astrocytic branchlets (magenta) and leaflets (green). Color intensity is log-probability of the corresponding (H,C) pair to be found in the images, rescaled for better contrast. Black dots—overlap between the two color channels. Yellow curve: local median complexity for given entropy, taking all values (from branchlets and leaflets) together. Top, marginal distributions of entropy, kernel density estimate. Right, marginal distributions of complexity, kernel density estimate. **(C)** Distributions of “excess complexity,” i.e., difference between C values and local median curve, kernel density estimate.

The spatial organization of leaflets and branchlets was then analyzed using the complexity-entropy spectrum analysis. To estimate local complexity and entropy, we redefined the probability measure used by Zunino and Ribeiro (Ribeiro et al., 2012; Zunino and Ribeiro, 2016). Instead of using permutation entropy (Bandt and Pompe, 2002), we used local statistics of shearlet transform coefficients (Kutyniok and Labate, 2012). The analysis was performed on mean projections along the Z-axis of the segmentation stacks either of astrocytic leaflets or of astrocytic branchlets (Figure 5A). Well-structured astrocytic branchlets had low entropy and low complexity, while the space between the branchlets covered a wider spectrum of entropy-complexity pairs (H,C) (Figure 5A). The astrocytic leaflets formed a more chaotic image, which is reflected in higher entropy values. The presence of coincidental anisotropic spatial features across several spatial scales accounted for zones of higher spatial complexity (Figure 5A).

Thus, branchlets and leaflets followed distinct complexity-entropy spectra (Figure 5B): (1) (H,C) pairs for branchlets extended to the area of low entropy and of low complexity, while (H,C) pairs for the leaflets reached higher maximal complexity and were clustered in the area of higher entropy and of higher complexity; (2) in the areas where the two distributions overlapped, the branchlets tended to have higher complexity for the same entropy values than leaflets. These differences become more evident if one compares deviations of complexity values from the median curve calculated for the pooled (H,C) values from the two groups (Figure 5C). Most of the (H,C) values for leaflets were below the median curve (negative deviations), whereas most values for branchlets were above the curve (positive deviations).

**Figure 6:**
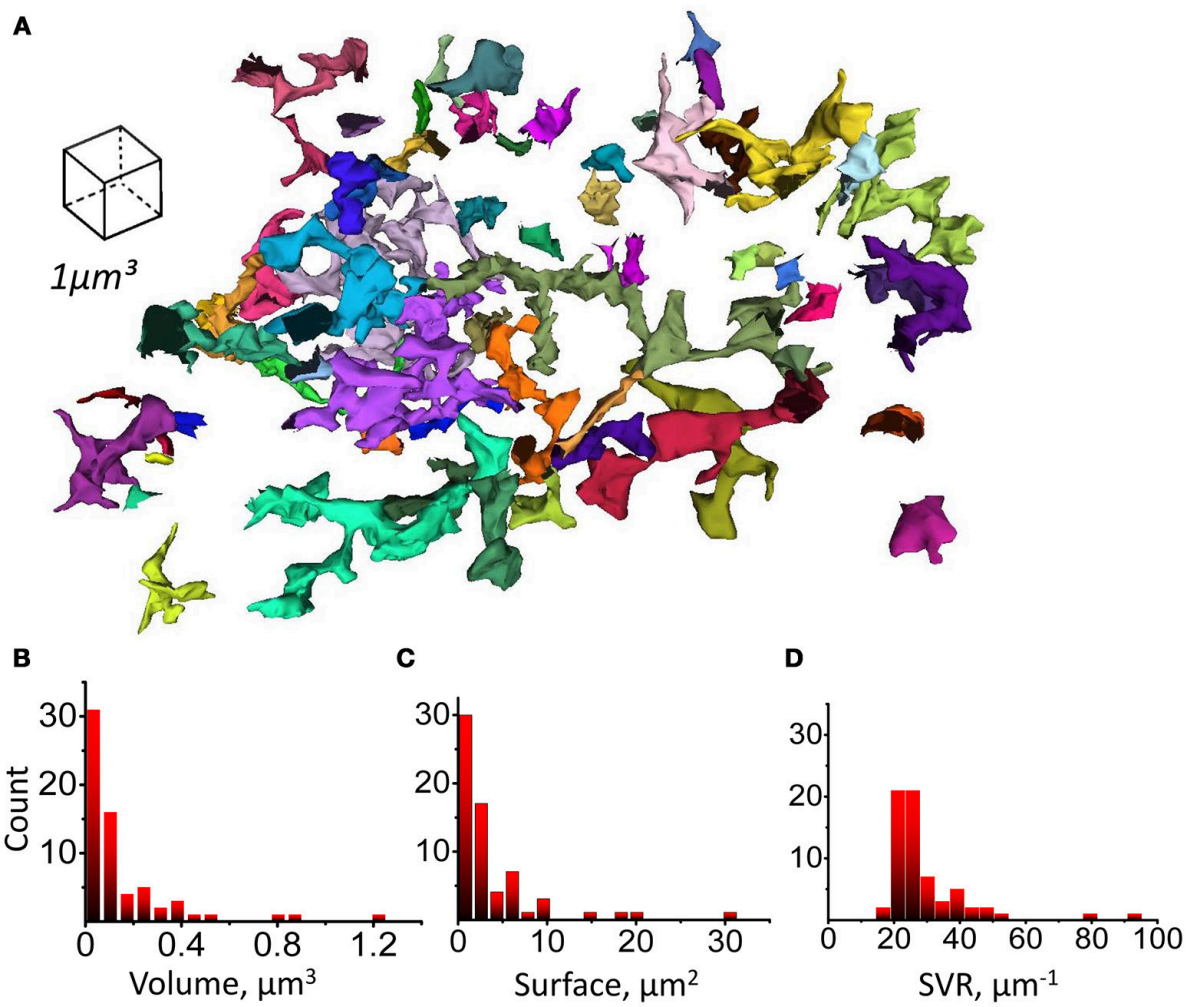
Leaflets parameters distributions. **(A)** Complete (uncut) structures identified as leaflets according to their morphological properties and having a connection to parent branchlet considered as individual leaflets. Whole leaflet volume, surface and SVR was measured. **(B)** Distribution of leaflet volumes. **(C)** Distribution of leaflet surfaces. **(D)** Distribution of leaflet SVRs.

Next, we tested if astrocytic leaflets can be classified according to their morphological properties likewise dendritic spines in neurons (Hering and Sheng, 2001). We segmented whole leaflets as complete astrocytic processes with high SVR, devoid of organelles and connected to parent astrocytic branchlet (Figure 6A). Volume, surface and SVR of whole individual leaflets were analyzed. All these parameters had skewed distributions without obvious peaks which would indicate the existence of different classes of the leaflets (Figures 6B–D). Therefore, the leaflets were not subdivided further in this study. However, further studies based on other parameters may suggest a classification of astrocytic leaflets. For example, the distribution of the distances from leaflet to its nearest neighbor leaflet on the same parent branchlet could be fitted with two Gaussian distributions with means of 0.23 and 0.58 μm (Supplementary Figure 5), but no correlation of the distance to the nearest neighbor with the leaflet volume was observed (Supplementary Figure 5).

### Asymmetric astrocytic coverage of axonal boutons and dendritic shafts

**Figure 7:**
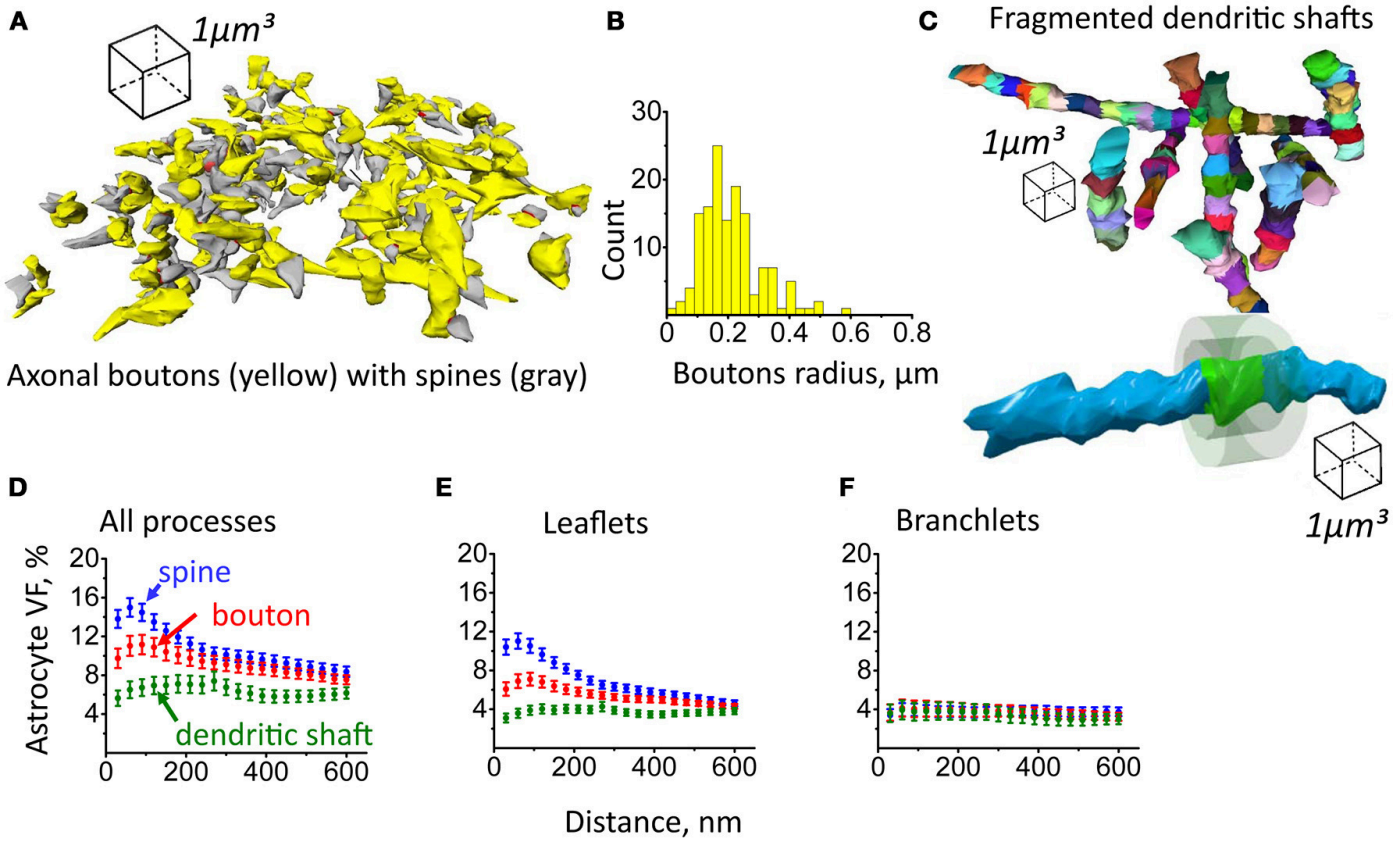
Astrocyte VF distribution around axonal boutons, dendritic shafts and dendritic spines. **(A)** 3D reconstruction of axonal boutons (yellow) and dendritic spines (gray). **(B)** Distribution of axonal boutons according to their radii. **(C)** Top, reconstruction of dendritic shafts. The shafts were fragmented to 1 μm long chunks. Bottom, equidistant surfaces were plotted around the side surface of each chunk. The VF of astrocytic processes was estimated between these surfaces. **(D)** Distribution of astrocyte VF with the distance from the spine (blue circles), axonal bouton (red circles) and dendritic shaft (green circles). **(E)** Distribution of leaflets VF with the distance from spine (blue circles), axonal bouton (red circles) and dendritic shaft (green circles). **(F)** Distribution of branchlets VF with the distance from spine (blue circles), axonal bouton (red circles) and dendritic shaft (green circles). The data on panels **(D–F)** are presented as mean ± SEM.

Next, axonal boutons and dendritic shafts were reconstructed. Consistent with previous studies, the distribution of boutons radii pointed to the diversity of these structures (De Paola et al., 2006; Hara et al., 2011; Grillo et al., 2013). However, further classification of axonal boutons was beyond the scope of this study, and they were treated as a single pool (Figures 7A,B). Dendritic shafts were fragmented into 1 μm long chunks which were approximately in the size range of dendritic spines and axonal boutons (Figure 7C). We then generated equidistant surfaces around each bouton and chunk of dendritic shaft to estimate astrocyte VF distribution around these structures in comparison to dendritic spines. The highest VF_max_ of astrocyte processes was found around dendritic spines (VF_max_: 19.2 ± 0.9%, *n* = 207, Figure 7D; Note, that VF_max_ was located at a different distance from each spine; therefore, mean VF_max_ is higher than the peak of the mean VF in Figure 7D). VF_max_ of astrocyte processes around axonal boutons was significantly smaller (VF_max_: 15.6 ± 0.97%, *n* = 139, *p* = 0.007 for difference with spines, Mann-Whitney test, Figure 7D). Such asymmetry of glia near central synapses has been suggested as a mechanism favoring glutamate spillover onto presynaptic autoreceptors, which are responsible for activity-dependent regulation of glutamate release probability (Lehre and Rusakov, 2002). VF_max_ of astrocytic processes around dendritic shaft was smallest (VF_max_: 11 ± 1%, *n* = 96, *p* < 0.001 for difference with spines, *p* < 0.001 for difference with boutons, Mann–Whitney test, Figure 7D). These results suggest that astrocytic processes preferentially approach synaptic structures, leaving the dendritic shaft less covered. Such arrangement of the processes may not only serve to increase the efficiency of glutamate uptake and K^+^ clearance but also to isolate synapse from the extrasynaptic glutamate (Lozovaya et al., 2004; Wu et al., 2012). On the other hand, lower coverage of dendritic shafts by astrocytic processes will make glutamate uptake less efficient around this compartment, and this will facilitate activation of extrasynaptic receptors by extracellular glutamate.

### Astrocytic leaflets but not branchlets form PAP

Previously, we have already reported that hippocampal synapses are covered by organelle-free leaflets but not by astrocytic branchlets containing Ca^2+^ stores (Patrushev et al., 2013). Here we compared the distribution of astrocytic leaflets and of astrocytic branchlets around axonal boutons, dendritic spines and dendritic shafts. Interestingly, different VF_max_ of astrocyte around these neuronal compartments correlated with different VF_max_ of astrocytic leaflets (VF_max_ for spines: 15 ± 1%, *n* = 207; VF_max_ for boutons: 10.6 ± 0.7% *p* < 0.001 for difference with spines; VF_max_ for dendritic shafts: 7.4 ± 0.6%, *n* = 96, *p* < 0.001 for difference with spines, *p* < 0.001 for difference with boutons; Mann–Whitney test, Figure 7E). There were no compartment-specific differences in VF_max_ of astrocytic branchlets (VF_max_ for spines: 5.6 ± 0.7%, *n* = 207; VF_max_ for boutons: 6.3 ± 0.9% *p* = 0.96 for difference with spines; VF_max_ for dendritic shafts: 4.6 ± 0.8%, *n* = 96, *p* = 0.9 for difference with spines, *p* = 0.31 for difference with boutons; Mann–Whitney test, Figure 7F). This finding suggests that astrocytic branchlets host leaflets that extend toward synapses to cover them.

**Figure 8:**
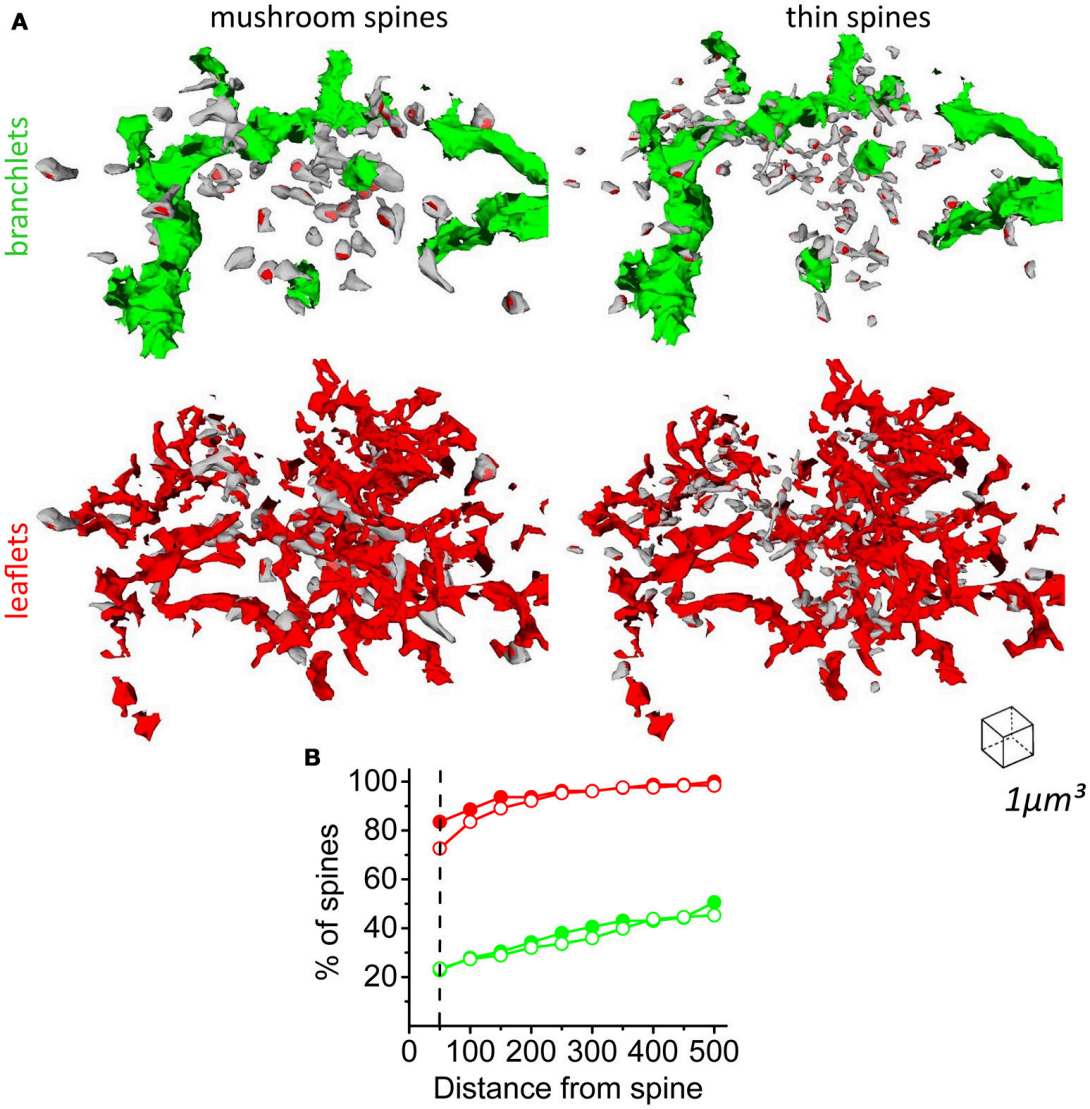
Distribution of mushroom and thin spines around astrocytic branchlets and leaflets. **(A)** Top left, distribution of mushroom spines (gray) around astrocytic branchlets (green). Top right, distribution of thin spines (gray) around astrocytic branchlets (green). PSDs – red. Bottom left, distribution of mushroom spines around leaflets (red). Bottom right, distribution of thin spines around leaflets (red). **(B)** Percentage of thin (empty circles) and mushroom (filled circles) spines that have leaflets (red lines) or astrocytic branchlets (green traces) at different distances. Dashed line corresponds to 50 nm distance, which was considered a direct contact between spine and astrocytic process.

Because leaflets are devoid of organelles, including Ca^2+^ stores (ER and mitochondria), they cannot support Ca^2+^ release from endogenous Ca^2+^ stores as means of neuron-astrocyte signaling (Li and Rinzel, 1994; Agarwal et al., 2017; Sherwood et al., 2017). Nevertheless, there is a possibility that some synapses are located close to the astrocytic branchlets containing Ca^2+^ stores. We found that both thin and mushroom dendritic spines are predominantly located within the area of the leaflets, but not in proximity to astrocytic branchlets (Figure 8A). 72.7% of thin spines and 83.5% of mushroom spines had direct contact with leaflets, but only 22.8% of thin spines and 23.4% of mushroom spines had direct contact with the astrocytic branchlets (Figure 8B). This finding suggests that the majority of glutamate synapses cannot directly trigger Ca^2+^ release from endogenous Ca^2+^ stores; however, it does not rule out that they trigger Ca^2+^ entry through plasma membrane in leaflets (e.g., due to glutamate uptake-dependent activation of Na^+^/Ca^2+^ exchanger) (Reyes et al., 2012; Bazargani and Attwell, 2016).

## Discussion

Astrocytic coverage of synapses plays an important role in shaping synaptic transmission and neurotransmitter spillover in the brain. We used three different methods to estimate the distribution of astrocyte VF around dendritic spines in CA1 hippocampal neuropil: from the center of mass of the PSD, from the edge of PSD and from the spine surface. When measured from the center of mass or from the edge of the PSD, the maximum astrocyte VF appeared closer to the PSD of smaller spines. However, astrocyte VF around the spine surface did not depend on the spine radius. This finding suggests that the spine size does not actually determine the astrocytic VF in it's microenvironment, it was previously suggested (Lushnikova et al., 2009; Bernardinelli et al., 2014a; Medvedev et al., 2014). When the spine size increases, it simply pushes the PAPs further away from the PSD and presynaptic active zone. This also leads to smaller and slower extrasynaptic glutamate concentration profiles. The net effect is a progressive reduction in the activation of extrasynaptic receptors around larger synapses, particularly pronounced for GluN receptors. This finding has multiple intriguing implications because it suggests that: (1) activation of extrasynaptic receptors because of glutamate spillover is more likely to occur around smaller synapses; (2) smaller spines are more likely to undergo long-term plasticity dependent on extrasynaptic GluN receptor activation; (3) larger spines are optimized for point-to-point communication, whereas smaller spines also mediate extrasynaptic signaling. Thus, the ratio between thin and mushroom spines shapes the propensity of a neuron to communicate through point-to-point or volume transmission in the hippocampus. In addition, larger spines have a lower SVR. A similar density of PAPs at small and larger spines may lead to reduced metabolic support per unit of cytoplasmic volume. This can potentially limit metabolic processes in larger spines relatively to thin spines, and thus serve as another plasticity limiting factor (Magistretti, 2011).

Astrocytic processes are morphologically highly plastic structures (Genoud et al., 2006; Theodosis et al., 2008; Tanaka et al., 2013; Bernardinelli et al., 2014a; Heller and Rusakov, 2015). Our findings suggest that the morphological plasticity of astrocytic processes may not be directly related to morphological plasticity of the associated synapses. Further studies are required to distinguish between different possibilities such as: (1) astrocytic morphological plasticity is not specific to particular synapses but is associated to groups of local synapses; or (2) it is synapse specific but is triggered independently of morphological plasticity of the spines. Alternatively, PAPs can regulate synaptic morphological plasticity themselves. For example, a contact with PAPs inhibits spine enlargement during memory consolidation in the amygdala (Ostroff et al., 2014). In fact, morphological remodeling is only one form of plasticity in PAPs. Two-fold increase in glutamate transporter density has been reported in mouse cortical astrocytes following whisker stimulation (Genoud et al., 2006).

The astrocytic processes can be categorized into primary branches, secondary branchlets (rod-like processes containing organelles, including ER and mitochondria) and leaflets (thin sheet-like organelle-free compartments) (Fernandez et al., 1984; Patrushev et al., 2013; Khakh and Sofroniew, 2015). This organization is reminiscent of that of spiny neuronal dendrites: astrocytic leaflets protrude from branchlets just like spines protrude from dendritic shafts. By using a *K*-means algorithm, we distinguished leaflets and branchlets based on their local SVR. Then we compared the spatial organization of astrocytic branchlets and leaflets using a spatial complexity-entropy spectra analysis. Because leaflets have different shapes and orientations, their 2D projection is characterized by a higher entropy than that of branchlets. In fact, branchlets introduced areas with a preferred characteristic scale and orientation, which ultimately lowered the entropy values. Interestingly, regions with similar entropy levels showed higher complexity values for branchlets than leaflets. This can be explained by the presence of a wider range of spatial scales in branchlets, whereas leaflets all tend to be small. The entropy-complexity spectrum analysis is a useful method not only to compare properties of astrocytic branchlets and leaflets, but also to characterize changes in astrocytic processes associated with different physiological and pathological states (Plata et al., 2018). In the future, this method of analysis can be extended to structures related to other cell types, such as dendrites and locations of synapses, and subcellular features, such as ER, mitochondria, vesicles.

Leaflets had skewed distributions of volume, surface area and SVR without obvious peaks. Thus, we did not classify the astrocytic leaflets into subclasses. However, we detected two distinguishable peaks when analyzing the distribution of distances to the nearest neighboring leaflets along an astrocytic branchlet. There was no correlation between these distance values and the size of the analyzed leaflet. The second peak roughly matched the diameter of dendritic spines. We speculate that leaflets grow toward spines to form astrocytic cradle around them; therefore, neighboring dendritic spines can space leaflets along the astrocytic branchlet in the hippocampal neuropil.

Dendritic spines are approached by axonal terminals to form a two-partite synapse. Astrocytic leaflets approach the synapses to form an astrocytic cradle (Verkhratsky and Nedergaard, 2014). Our findings show that the leaflet VF is larger around the spine surface than around axonal boutons. This result confirms previous report from 2D EM analysis, showing an asymmetric arrangement of PAPs around hippocampal pre- and post-synaptic terminals (Lehre and Rusakov, 2002). Interestingly, we could not detect a peak of astrocyte VF around the dendritic shaft. The reason why astrocytic leaflets do not extend toward the dendritic shaft is not entirely clear. Dendritic spines are located sparsely enough to form an “impenetrable wall” for the thin leaflets. However, the dendrite surface can be occupied by shaft synapses (e.g., GABAergic), limiting the space available for the leaflets (Megías et al., 2001). Nevertheless, two conclusions can be made from our results. *First*, in the hippocampal neuropil, astrocytic leaflets are specialized structures that form an astrocytic cradle around glutamatergic synapse. This cradle is asymmetric and has an opening on the presynaptic side. This structure might ensure glutamate and K^+^ clearance from the synapse and prevent the entry of extrasynaptic glutamate into the synaptic cleft (Lozovaya et al., 2004; Wu et al., 2012). *Second*, the low VF of astrocytic leaflets near dendritic shaft suggests less efficient neurotransmitter uptake around this compartment. The dendritic shaft contains extrasynaptic receptors and shaft synapses. This finding is consistent with lack of astrocytic cradle around shaft synapses, which may facilitate neurotransmitter spillover and activation of dendritic extrasynaptic receptors. Because many shaft synapses are GABAergic in CA1 pyramidal neurons, this may be a morphological mechanism promoting the activation of tonic GABA_A_ conductances through lower astrocytic GABA uptake along the dendritic shaft (Kersante et al., 2013; Song et al., 2013). Unlike glutamate receptors, GABA receptors are not permeable to K^+^ (Wollmuth and Sobolevsky, 2004; Shih et al., 2013; Cheung et al., 2015). For this reason, GABAergic synapses may not require the presence of PAPs for K^+^ clearance.

Since astrocytic leaflets have no ER or mitochondria, they can only generate Ca^2+^ transients via Ca^2+^ entry through plasma membrane (Zorec et al., 2012; Bazargani and Attwell, 2016). Astrocytic branchlets have these intracellular organelles and can therefore generate/amplify Ca^2+^ signals via Ca^2+^ release from internal stores. Several reports have suggested that this is one the major signaling pathways to ensure neuron-astrocyte communication (Ullah et al., 2006; Agarwal et al., 2017; Sherwood et al., 2017). However, our morphological analysis indicates that less than a quarter of all synapses contribute to this form of signaling in hippocampal CA1 *str.radiatum*. This does not rule out that remote synapses can still modulate Ca^2+^ dynamics in astrocytic branchlets via glutamate spillover. Glutamate escaping synapses can reach high-affinity mGluRs on an astrocytic branchlet and trigger IP_3_ production. IP_3_ can also be generated in leaflets in response to local synaptic activity and then diffuse intracellularly to the astrocytic branchlet. Likewise, Ca^2+^ entering leaflets through plasma membrane can diffuse intracellularly to the parent branchlet. This scheme portrays the astrocytic branchlet with leaflets similarly to the dendritic shaft with dendritic spines. Dendritic spines receive synaptic signals (electric and Ca^2+^) which are integrated and amplified (dendritic spike) in the parent dendritic shaft (Larkum and Nevian, 2008; Spruston, 2008; Takahashi et al., 2012). Leaflets receive synaptic signals which are integrated and amplified in the parent astrocytic branchlet. This hypothesis, however, requires further experimental testing and mathematical modeling. Similar methods used for dendritic integration studies (e.g., local glutamate uncaging) can be employed to study the integration of Ca^2+^ signals in the astrocytic branchlet.

Most of modern cellular neuroscience methods have their caveats: Whole-cell recordings dialyze the cytoplasm preventing some forms of signaling and plasticity. Fluorescent Ca^2+^ sensors work as buffers and, therefore, strongly affect Ca^2+^ dynamics. Voltage-sensitive dyes affect the cell membrane properties, hence, the excitability of the cells. Super-resolution optical imaging requires intense laser illumination which increases local temperature and may affect metabolic processes. Mathematical models are based on numerous assumptions and simplifications. Overall, validity of animal research for understating human brain function is often questioned. Chemical fixation used in this study considered as a gold standard in the field for decades. Nevertheless, it affects the size and the structure of the extracellular space and distorts the morphological relationship between spines and astrocytic processes (Kinney et al., 2013; Korogod et al., 2015; Nicholson and Hrabetová, 2017). The use of electron microscopy to study extracellular space therefore has significant limitations. Thus, a biophysical approach based on diffusion should be used as a method of choice (Nicholson and Hrabetová, 2017). Although distortions in the neuropil happen because of chemical fixation, they likely affect the microenvironment around small and large spines to the same extent.

Finally, our conclusions are only applicable to the astrocytic coverage of the glutamatergic synapses in CA1 hippocampal neuropil. The scenario could differ in other brain regions: astrocytes cover 74% of the cerebellar Purkinje cell spines and 29% of the dendritic spines in the mouse visual cortex (Spacek, 1985; Ventura and Harris, 1999). In addition, astrocytic coverage of GABAergic synapses was not addressed in this work. However, recent reports highlighted important differences in the regulation of synaptic and extrasynaptic GABAergic signaling by neuronal and astrocytic GABA transporters (Kersante et al., 2013; Song et al., 2013). Therefore, the astrocytic coverage of GABAergic synapses also requires further morphological analysis.

## Ethics statement

All animal experiments have been performed with approval of Nizhny Novgorod State University ethics committee.

## Author contributions

NG and IG performed the 3D reconstruction; NG, ASc, and AB analyzed the data and prepared the figures; ASc performed the diffusion simulations; VT discussed the results and consulted on the analysis; ASe, ASc, and AB wrote the paper; ASe supervised the project, planned the analysis, prepared the figures.

### Conflict of interest statement

The authors declare that the research was conducted in the absence of any commercial or financial relationships that could be construed as a potential conflict of interest. The reviewer, CC and the handling Editor declared their shared affiliation.
