# Supplementary material for: Astrocytic Coverage of Dendritic Spines, Dendritic Shafts, and Axonal Boutons in Hippocampal Neuropil
Source: Front Cell Neurosci. 2018 Aug 17;12:248. doi: 10.3389/fncel.2018.00248 (PMC6108058; doi:10.3389/fncel.2018.00248)
Supplement: Supplementary file 2 [file Data_Sheet_1.PDF]

Astrocytic coverage of dendritic spines, dendritic shafts and axonal boutons in hippocampal neuropil

Nikolay Gavrilov, Inna Golyagina, Alexey Brazhe, Annalisa Scimemi, Vadim Turlapov and Alexey Semyanov

Supplementary information

Entropy and statistical complexity

A spatial pattern can be characterized by a pair of statistical properties, namely entropy and statistical complexity (Ribeiro et al., 2012). Intuitively, a highly ordered, crystalline structure with a single spatial scale and preferred feature orientation will have both low entropy and small statistical complexity, as the local structure is the same everywhere. At the other end of the complexity-entropy spectrum, where the pattern is totally random with no spatial correlations, the entropy of the system will be high, while the complexity will again be low (the spatial pattern has the same local statistics). Intermediate cases with high statistical complexity are of more interest, as they represent systems with non-trivial regularities and underlying structure. We developed an algorithm to map local entropy and complexity values for biologically relevant structures using shearlet transform to induce local probability densities of scale and orientation and Jensen-Shannon divergence to define statistical complexity. Below we describe the two points in more detail.

Both entropy and complexity (entropic non-triviality) measures must rely on distribution of spatial features, such as local prevalence of some scale or orientation

$P := \{P_i\}$. (S1)

Then entropy can be defined simply as Shannon information entropy

$S[P] = -\sum_i P_i \, log_2 P_i$. (S2)

Entropy will have its maximum for the equiprobable distribution of all features

$P := \{P_i\}_e, \; S[P_e] = S_{max} = log_2 N,$ (S3)

where N is number of possible states or features. This allows us to introduce normalized entropy:
$H\_s[P]: S[P]/S[P\_e]$, $H_s[P] \; \in [0, \ldots 1]$. (S4)

Following (Lamberti et al., 2004) we use the disequilibrium-based complexity measure

$$C[P] := Q_{JS}[P, P_e]H_s[P], \quad \text{(S5)}$$

i.e. the one based on statistical distance between the observed (P) and equiprobable (P_e) distributions. Here, following (Rosso et al., 2007), we employ normalized Jensen-Shannon divergence

$$Q_{JS} = J[P, P_e]/J_{max} \quad \text{(S6)}$$

as a measure of distance between two distributions, where Jensen-Shannon divergence is defined as $J[P, P_e] = S[\frac{P+P_e}{2}] - \frac{1}{2}(S[P] + S[P_e])$. (S7)

Clearly, $J[P, P_e] = 0$ if $P = P_e$ and reaches its maximum when only one feature, say m, is present, while all others are absent: $P_i = 1 \mid i = m$, and $P_i = 0 \mid i \neq m$.

Shearlet transform

Entropy and complexity measures are defined as functionals of some spatial probability density function. Let us see how shearlet transform can provide a convenient density function, describing the local prevalence of structures with some specific scale and orientation. We use fast finite discrete shearlet transform (FFST) described in detail by (Häuser and Steidl, 2013). Here we provide a minimally sufficient description of the FFST required for our definition of spatial entropy and complexity.

Discrete shearlet transform is based on convolving the digital 2D image $I \in R^{(N,N)}$ with scaled, sheared, and shifted copies of a "mother" shearlet function ψ, thus accounting for different scales and orientations of features contained in the image; one uses the dilation matrix A_a and shear matrix S_s,

$$A_a = \begin{pmatrix} a & 0 \\ 0 & \sqrt{a} \end{pmatrix}, a \in \mathbb{R}^+; S_s = \begin{pmatrix} 1 & s \\ 0 & 1 \end{pmatrix}, s \in \mathbb{R} \quad \text{(S8)}$$

This results in sheared, scaled and shifted copies of the mother wavelet

$$\psi(x) : \psi_{a,s,t} = a^{-3/4}\psi({A_a}^{-1}{S_s}^{-1}(x - t)). \quad \text{(S9)}$$

An example of mother shearlet function ψ is shown in Supplementary Figure 3. It is markedly anisotropic, with a higher spatial resolution across its main axis than along it. Thus, the scaled and

shared copies of ψpick up dominant anisotropic features at different spatial scales and orientations. In the discrete transform, one uses a fixed number of decomposition scales and shifts as well as scale-dependent number of orientations (more orientations at higher spatial frequencies). Finally, shearlet decomposition of image I is given by shearlet coefficients

$$T(I)(j,k,m) = \langle I, \psi_{i,k,m} \rangle \ , \tag{S10}$$

where discrete shearlet $\psi_{i,k,m} = \psi_{a_i s_{j,k} t_m}(x)$ is the shearlet at discrete scale a_j, shear $s_{j,k}$ and shift t_m. Thus, $T(I)$ is a set of K images of the same size as I, where the value at a specific (x,y) location in the k-th image represents the shearlet coefficient at some specific scale j and shear s.

Following ideas from wavelet entropy and earlier of spectral entropy of *(Powell and Percival, 1979)*, in each location of the studied 2D image $I(x,y)$, we define

$$P(x,y) = \{P_k(x,y)\} \tag{S11}$$

as the normalized power of the shearlet coefficients

$$E_k(x,y) = \sigma_k \star T(x,y)^2 \tag{S12}$$

at this point:

at this point:

$$P_k(x,y) = E_k\,(x,y)/\textstyle\sum_j E_j\,(x,y) \tag{S13}$$

thus interpreting a spectrum of local feature scales and orientations as a density function. Here $\sigma_k \star\cdot$ denotes convolution with a Gaussian kernel with scale-dependent standard deviation $\sigma_k = 2^j\sigma$.

References

Häuser, S., and Steidl, G. (2013). Convex multiclass segmentation with shearlet regularization. *International Journal of Computer Mathematics* 90**,** 62-81.

Lamberti, P.W., Martin, M.T., Plastino, A., and Rosso, O.A. (2004). Intensive entropic non-triviality measure. *Physica A: Statistical Mechanics and its Applications* 334**,** 119-131.

Powell, G.E., and Percival, I.C. (1979). A spectral entropy method for distinguishing regular and irregular motion of Hamiltonian systems. *Journal of Physics A: Mathematical and General* 12**,** 2053.

Ribeiro, H.V., Zunino, L., Lenzi, E.K., Santoro, P.A., and Mendes, R.S. (2012). Complexity-entropy causality plane as a complexity measure for two-dimensional patterns. *PLoS One* 7**,** e40689.

Rosso, O.A., Larrondo, H.A., Martin, M.T., Plastino, A., and Fuentes, M.A. (2007). Distinguishing Noise from Chaos. *Physical Review Letters* 99**,** 154102.

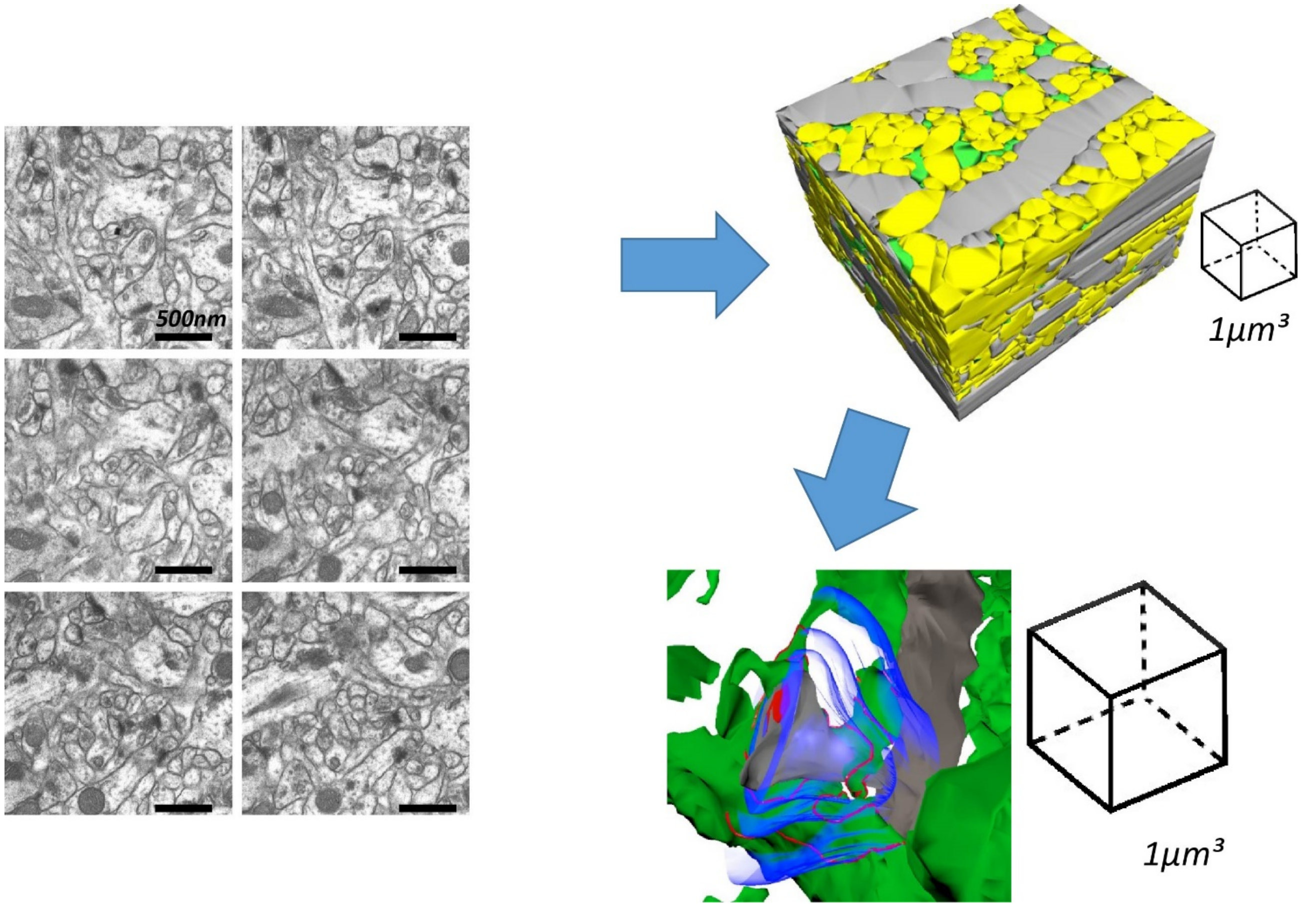

Supplementary Figure 1. 3D reconstruction and analysis of electron microscopy images

Schematics describing the procedure of astrocyte VF analysis. First, electron microscope serial sections of CA1 *str.radiatum* block were aligned. Cellular structures such as axons, dendrites, dendritic spines, astrocytic processes, etc., were visually identified and traced. Second, full block reconstruction was done with Reconstruct software written by John C. Fiala. Dendritic shafts and dendritic spines – grey; astrocytic processes – green; axons – yellow. Notably, axons occupied most of the volume in this region. Third, equidistant surfaces were constructed around structures of interest (e.g. center of PSD, surface of PSD, surface of dendritic spine, surface of presynaptic bouton, side surface of dendritic shaft chunk). The surfaces were equally spaced and were used for analysis of distribution of astrocytic VF.

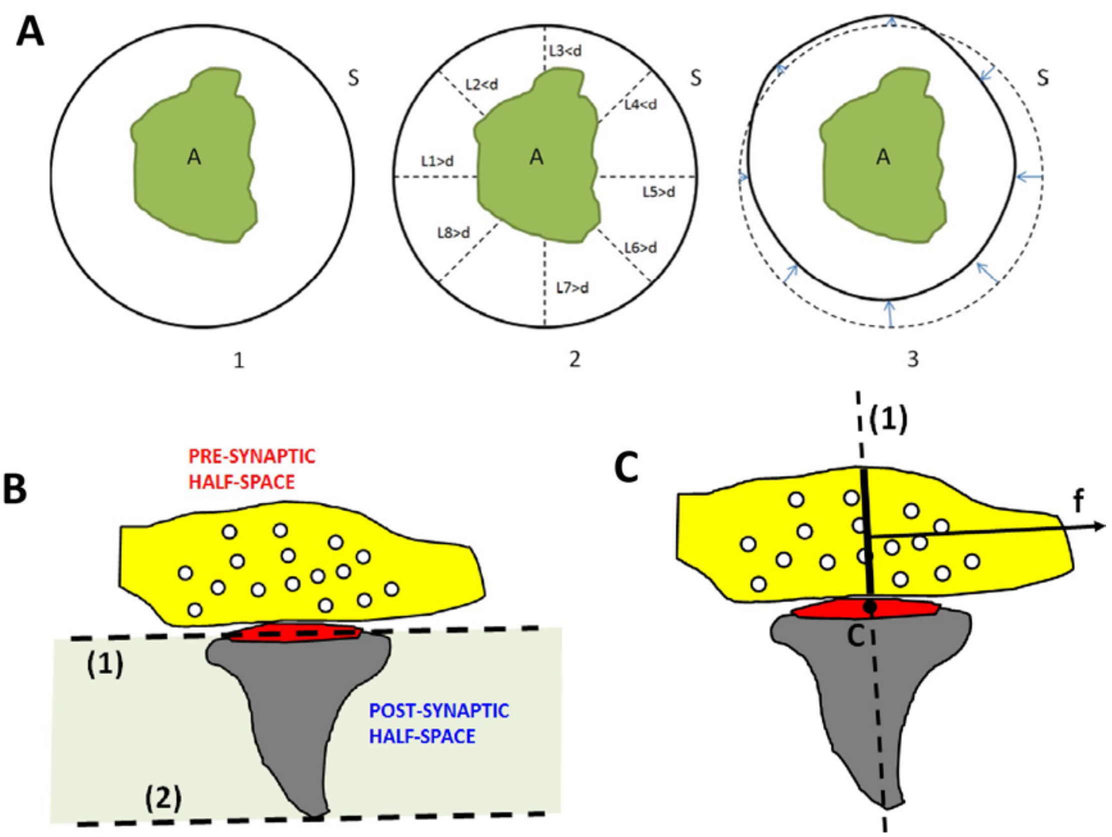

Supplementary Figure 2. Equidistant surface analysis

A. Equidistant surface generation: (1) A sphere which encloses an object *A* was generated. The sphere was triangulated. (2) The shortest distance to *A* was calculated for each vertex of the triangulated sphere. *L1, L2, …* – are distances for different vertexes, *d* – desired distance. (3) If *Li>d* then the vertex was moved towards *A.* Otherwise the vertex was moved further from *A*. **B.** The 'presynaptic half space' was formed by plane (1). The plane intersects PSD center of mass and has minimal sum:

$$\sum |dot(center - p_i, normal)|,$$

where p_i is a PSD surface vertex, *normal* is a normal vector for the plane, *center* is the PSD center of mass, *dot* is the dot product of vectors. The plane (2) was parallel to plane (1) and intersected the spine in the vertex, most distant from plane (1). Assuming the dendrite beneath plane (2), the corresponding half-space was named 'dendritic half-space'. **C.** Axonal bouton radius calculation: Vector *f* for the bouton vertexes was used as a normal vector for the plane (1) that intersects PSD center *C*. Intersection between plane (1) and axonal bouton gave a contour which was used for axonal bouton thickness estimation. The radius of the circle which circumscribed the contour was considered as a radius of axonal bouton.

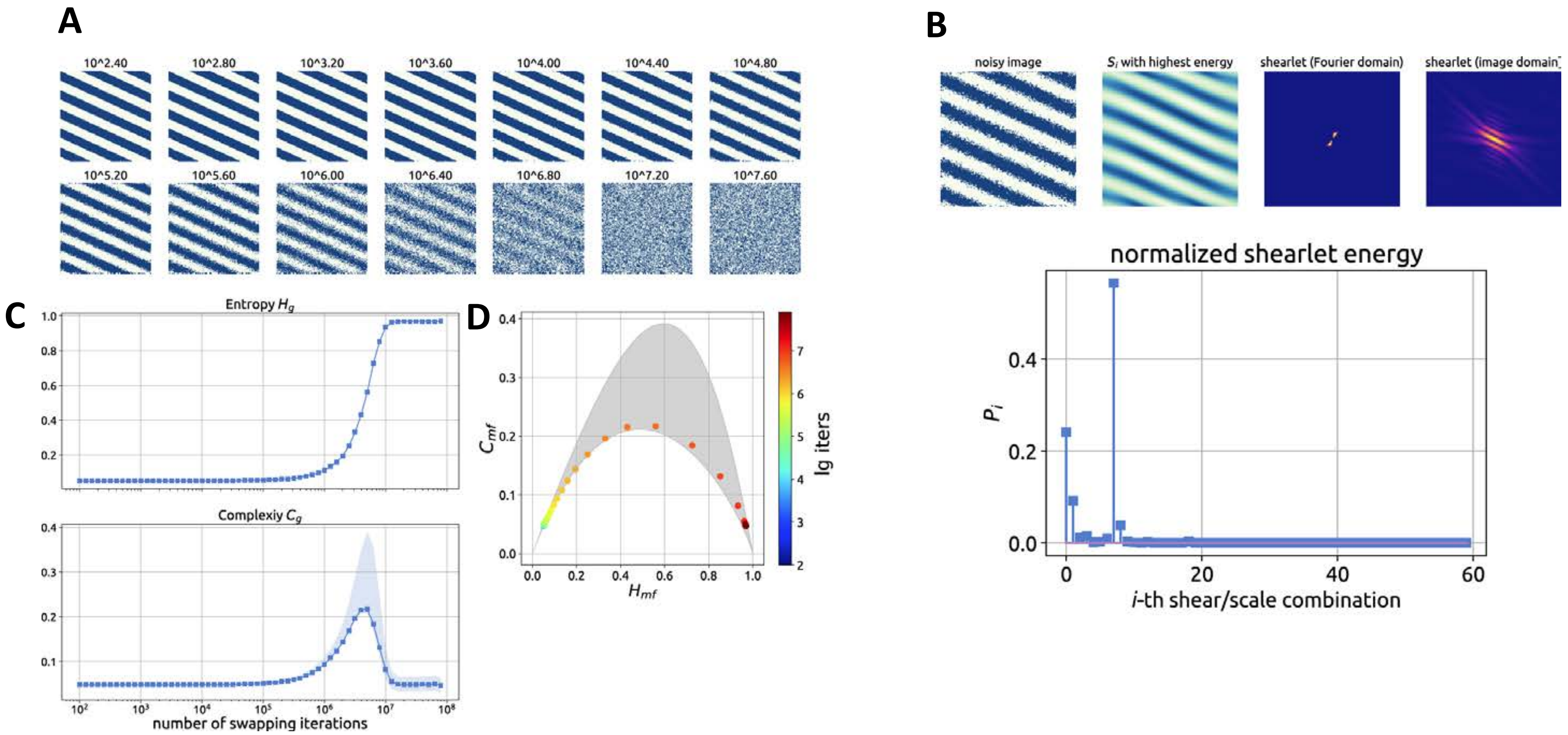

Supplementary Figure 3. The complexity-entropy analysis illustrated on a toy example of radially corrupted stripe pattern

A. Example spatial patterns with gradually increasing entropy. *P* values on top represent probability for each pixel in the image to have its intensity value swapped with some randomly chosen other pixel. **B.** Example of shearlet power spectrum, shearlet coefficients with the highest power, and the corresponding shearlet function in Fourier and spatial domains. **C.** Increasing the probability of pixel swap results in monotonic increase in entropy and a peaking dependence for spatial complexity. **D.** The complexity-entropy spectrum of the images for different pixel swap probabilities.

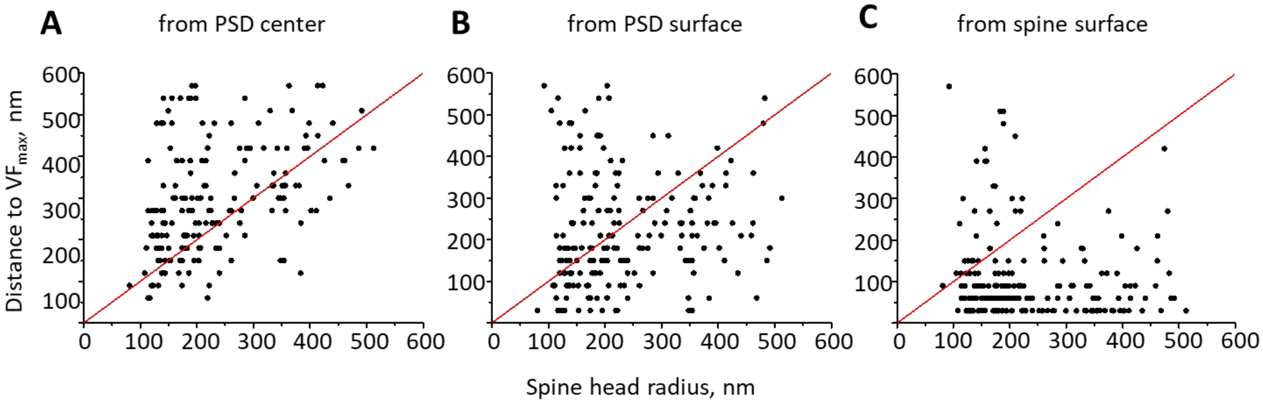

Supplementary Figure 4. Spine borders push astrocyte processes from the center of PSD

Relation between spine head radius and distance to astrocyte VF peak: **A.** measured from PSD center, **B.** measured from PSD surface and **C.** measured from spine surface. When the VF_{max} was measured from the PSD center, the distance to VF_{max} was roughly proportional to the spine radius (*red line*). When the VF_{max} was measured from the spine surface, the distance to VF_{max} did not depend on spine size. Thus, spines of all sizes enwrapped by astrocyte processes similarly and only spine size determines how far these processes are located from the center of PSD, e.g. presynaptic active zone.

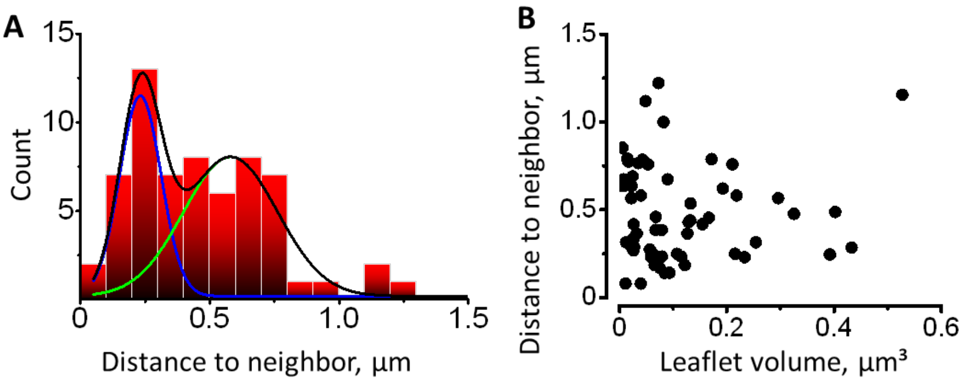

Supplementary Figure 5. Distribution of leaflets along parent astrocytic branch

A. Distribution of distances to neighboring leaflet. It can be fit by the sum of two Gaussian distributions. **B.** The relationship between distance to the neighboring leaflet and the volume of analyzed leaflet.
